# The Effects of Epidermal Neural Crest Stem Cells on Local Inflammation Microenvironment in the Defected Sciatic Nerve of Rats

**DOI:** 10.3389/fnmol.2017.00133

**Published:** 2017-05-22

**Authors:** Yue Li, Dongdong Yao, Jieyuan Zhang, Bin Liu, Lu Zhang, Hua Feng, Bingcang Li

**Affiliations:** ^1^Department of Neurosurgery, Southwest Hospital/State Key Laboratory of Trauma, Burns and Combined Injury, Third Military Medical UniversityChongqing, China; ^2^Research Institute of Surgery, Daping Hospital/State Key Laboratory of Trauma, Burns and Combined Injury, Third Military Medical UniversityChongqing, China; ^3^School of Life Sciences/Key Laboratory of Freshwater Fish Reproduction and Development of Education Ministry, Southwest UniversityChongqing, China; ^4^Children’s Hospital of Chongqing Medical University/Ministry of Education Key Laboratory of Child Development and Disorders, Chongqing Medical UniversityChongqing, China

**Keywords:** EPI-NCSCs, cell transplantation, sciatic nerve injury, macrophage phenotypes, inflammatory cytokines

## Abstract

Cell-based therapy is a promising strategy for the repair of peripheral nerve injuries (PNIs). epidermal neural crest stems cells (EPI-NCSCs) are thought to be important donor cells for repairing PNI in different animal models. Following PNI, inflammatory response is important to regulate the repair process. However, the effects of EPI-NCSCs on regulation of local inflammation microenviroment have not been investigated extensively. In the present study, these effects were studied by using 10 mm defected sciatic nerve, which was bridged with 15 mm artificial nerve composed of EPI-NCSCs, extracellular matrix (ECM) and poly (lactide-co-glycolide) (PLGA). Then the expression of pro- and anti-inflammatory cytokines, polarization of macrophages, regulation of fibroblasts and shwann cells (SCs) were assessed by western blot, immunohistochemistry, immunofluorescence staining at 1, 3, 7 and 21 days after bridging. The structure and the function of the bridged nerve were determined by observation under light microscope and by examination of right lateral foot retraction time (LFRT), sciatic function index (SFI), gastrocnemius wet weight and electrophysiology at 9 weeks. After bridging with EPI-NCSCs, the expression of anti-inflammatory cytokines (IL-4 and IL-13) was increased, but decreased for pro-inflammatory cytokines (IL-6 and TNF-α) compared to the control bridging, which was consistent with increase of M2 macrophages and decrease of M1 macrophages at 7 days after transplantation. Likewise, myelin-formed SCs were significantly increased, but decreased for the activated fibroblasts in their number at 21 days. The recovery of structure and function of nerve bridged with EPI-NCSCs was significantly superior to that of DMEM. These results indicated that EPI-NCSCs could be able to regulate and provide more suitable inflammation microenvironment for the repair of defected sciatic nerve.

## Introduction

Peripheral nerves are playing an irreplaceable role in connecting the central nervous system with the sensory and motor organs, while their injury is still a very common clinical trauma that may lead to significant loss of sensory and motor functions (Li X. et al., 2010; Goldstein et al., 2016). Peripheral nerve injury (PNI) can induce complex pathophysiological processes and inflammatory response which are important in regulating the repair process of exacerbating tissue damage or promoting tissue repair (Siqueira Mietto et al., 2015). Inflammatory responses following PNI include increased vascular permeability, dynamic balance among inflammatory cytokines and extravasation of large numbers of neutrophils and monocytes/macrophages into the tissues (Gu et al., 2010).

Up to the present, the repair of peripheral nerve injury is still one of the most challenging concerns and tasks, especially for massive defect of peripheral nerve. As the development of tissue engineering, the biodegradable artificial nerve is an alternative to autologous nerve grafts and used to bridge the stumps of defected peripheral nerve. Poly lactic co-glycolic acid (PLGA) nerve conduit can be combined with different donor cells, such as neural stem cells (NSCs; Omi et al., 2016), bone marrow stromal cells (BMSCs; Ying et al., 2016), shwann cells (SCs; You et al., 2011), olfactory ensheathing cells (OECs; Guerout et al., 2014), epidermal neural crest stem cells (EPI-NCSCs; Zhang et al., 2014), to provide a favorable microenvironment for preventing infiltration of scar tissue and guiding axonal regeneration (Li B. C. et al., 2010).

EPI-NCSCs are neural precursor cells that retained the neurologic differentiation potential of their neural crest origin (Krejčí and Grim, 2010). Because of their advantages of physiological plasticity, multipotency, adequate source, easy access, no graft rejection and ethical issues, EPI-NCSCs are promising donor cells for the repair of nervous system injury (Pandamooz et al., 2013). Transplantation of EPI-NCSCs following spinal cord injury (SCI) can significantly improve touch perception and sensory connectivity by expressing extracellular proteases for modulating scar formation, neurotrophic factors for promoting cell survival and angiogenic factors for accelerating neovascularization (Sieber-Blum et al., 2006).

Appropriate inflammatory response is important for the healing of tissue injury. After PNI, inflammatory response is predominantly modulated by the infiltration and the polarization of macrophages (Chen et al., 2015), the activation of fibroblasts (Niapour et al., 2015) and SCs (Stratton et al., 2016), which is associated with a dynamic expression balance of pro- and anti-inflammatory cytokines. Macrophages have two different phenotypes, classically activated M1 macrophages possess neurotoxic effects, alternatively activated M2 macrophages promote damage repair and inhibit harmful immune responses (Fleetwood et al., 2007; Martinez et al., 2008; Kalampokis et al., 2013; Peluffo et al., 2015; Guan et al., 2016). Fibroblasts can induce inflammation, control the switch from acute to chronic inflammation, and play an important role in the attenuation of inflammation (Buckley et al., 2001).

In the past, EPI-NCSCs had been used to repair PNI (Amoh et al., 2009), but only a few studies were designed for probing into regulatory effects of EPI-NCSCs on inflammatory response after PNI (Stratton et al., 2016). In the present study, PLGA, EPI-NCSCs and extracellular matrix (ECM), an acellular component composed of laminin, proteoglycan and entactin which play an important role in maintaining the integrity of the tissue structure and providing a proper environment for donor cell adhesion and migration (Chernousov et al., 2008), were combined to fabricate artificial nerve for repairing sciatic nerve defect of rats. Besides the recovery of structure and function of the defected nerve, the local inflammation environment after bridging was specially observed. The present findings showed that EPI-NCSCs could significantly change the expression levels of pro- and anti-inflammatory cytokines, promote the polarization of macrophages from M1 to M2, and more properly regulate the activated fibroblasts and SCs, thus result in better improvement of the structure and function of the defected nerve.

## Materials and Methods

### Preparation of PLGA Conduits

**Figure 1:**
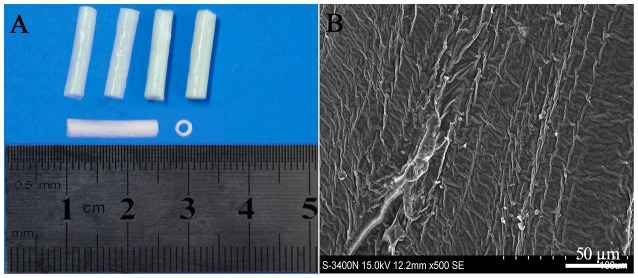
**Poly (lactide-co-glycolide) (PLGA) conduit (A)** and its structure under SEM **(B)**. Scale bar = 50 μm.

The PLGA conduits were made by a slight modification from Sundback et al. (2005). Briefly, 5% PLGA (PLA/PGA = 85/15, w/v, MW 15,000; Sigma, USA) solution in trichlormethane was gently mixed at 37°C for 24 h and pipetted into the glass culture dish to 0.5 mm high. After overnight evaporation, PLGA films in 25 μm thick were obtained, then rolled into the conduits with 14 layers by a glass capillary tube, and dried for 48 h. The dried PLGA conduits (length 15 mm, inner diameter 1.8 mm, outer diameter 2.5 mm, Figure 1A) had a rough surface and no big porous as confirmed by scanning electron microscope (Figure 1B). The PLGA conduits were sterilized by gamma irradiation (2.5 Mrad) and soaked in DMEM/F12 for 2 h before cell transplantation (Liu et al., 2008).

### Animals

All adult Sprague-Dawley rats (220–250 g, from Laboratory Animal Center Third Military Medical University, Chongqing, China) were housed care according to the guidelines of the Third Military Medical University (TMMU), and surgical procedures and post-operative care were conducted in accordance with protocols approved by the TMMU Institutional Animal Care and Use Committee.

### Preparation of EPI-NCSCs

EPI-NCSCs were freshly dissected out from whisker follicles of adult green fluorescence protein (GFP) transgenic Sprague-Dawley rats as green fluorescent donor cells were easy to identify in the host tissue, or normal Sprague-Dawley rats according to our previous description (Zhang et al., 2012). Briefly, whisker follicles were dissected, cleaned and cut on cavernous sinus and ring sinus to roll out the bulges, then placed onto collagen-coated culture plates for 30 min, finally added 85% Dulbecco’s Modified Eagle’s Medium (DMEM)/F12 (Hyclone, USA), 10% fetal calf serum (Gibco, USA), 2% B27 (Gibco, USA) and 10 ng/ml of basic fibroblast growth factor (Sigma, USA) to culture at 37°C in a 5% CO_2_ incubator, while the medium was renewed every 3 days. Within approximately 72 h, EPI-NCSC started to emigrate from explants. Four days after onset of emigration, the bulges were removed for preventing ingrowth of other epidermal stem cells and the adhering EPI-NCSCs were resuspended by trypsin (Hyclone, USA), placed in fresh collagen-coated plates at 1 × 10^4^ cells per 35-mm plate, and cultured for another 4 days. Before the usage, the cells were collected in DMEM/F12 and adjusted to 2.0 × 10^4^ cells/μl.

Cultured EPI-NCSCs were identified by rabbit anti SOX10 (1:1000, Abcam, USA) and mouse anti Nestin (1:100, Abcam, USA) primary antibodies, visualized with Alexa Flour 594 goat anti-rabbit IgG (1:500, Abcam, USA) and Cy5 goat anti-mouse IgG (1:300, Abcam, USA) secondary antibodies. The positive cells were observed by fluorescence inverted microscope and counted in three view fields selected randomly from each hole. The cell purity was 99% as determined by the ratio of Nestin-SOX10 double positive cells and GFP-cells in the same view field.

### Sciatic Nerve Injury and Bridging

Adult female Sprague-Dawley rats were anesthetized with 1% sodium pentobarbital solution (40 mg/kg). Under aseptic conditions, the right sciatic nerve was exposed and a 10 mm segment of sciatic nerve was removed, leaving a 15 mm defect after retraction of the nerve stumps, then was bridged with PLGA conduit filled with the mixture of DMEM/F12 and ECM (composed of laminin, collagen type IV, heparin sulfate proteoglycan, and entactin; the control group, DMEM/F12—12.5 μl, ECM—12.5 μl, *n* = 83) and the mixture of EPI-NCSCs and ECM (the experimental group, cell suspension—12.5 μl, 2.0 × 10^4^ cells/μl, ECM—12.5 μl, *n* = 83). Following PLGA conduit and the nerve stump was sutured with 8–0 stitches, muscles and skin were separately closed with 6–0 lines. After rats resuscitated from the anesthetization, they were housed in their cages as usual.

### Western Blotting

At 1, 3, 7 and 21 days postoperatively, the segments (20 mm) of sciatic nerve grafts were isolated for the purpose of including both nerve stumps in which the events of inflammation and regeneration mainly occurred at early phase (*n* = 3 rats/group/time point), then were homogenized, lysed, and normalized for protein concentration using the total protein extracted kit (Keygen, China). Equal amounts of protein were loaded into each well, subjected to 10% SDS-PAGE gels, transferred to PVDF membrane and then blocked. The membranes were incubated overnight at 4°C with rabbit anti-interleukin-4 (IL-4, 1:2500; Abcam, USA), rabbit anti-interleukin-6 (IL-6, 1:500; Abcam, USA), mouse anti-interleukin-13 (IL-13, 1:500; Abcam, USA), rabbit anti-tumor necrosis factor-α (TNF-α, 1:1000; Abcam, USA), mouse anti-actin (1:3000; Abcam, USA), then incubated for 2 h at 37°C with goat anti-rabbit or mouse IgG horseradish peroxidase-conjugated (HRP; 1:20,000; Abcam, USA). Proteins were visualized by using the enhanced chemi-luminescence (ECL) procedure (Amersham Biosciences, USA) and the intensity of each band was expressed relative to that of actin.

### Immunological Stain

At 7 days after bridging, under anesthetization the bridged rats were perfused with 4% paraformaldehyde, then the segments of sciatic nerve were removed as above, and post-fixed in the same fixative for 4 h, then overnight in 30% sucrose.

For immunohistochemical staining, the segments were fixed and embedded in paraffin. Five micrometer longitudinal sections were cut (*n* = 3 rats/group), then treated with 3% H_2_O_2_ and blocked with 5% bovine serum albumin in 0.01 M PBS for 30 min at room temperature. The sections were incubated overnight at 4°C with rabbit anti-IL-4 (1:200; Abcam, USA), rabbit anti-IL-6 (1:1000; Abcam, USA), mouse anti-IL-13 (1:100; Abcam, USA), rabbit anti-TNF-α (1:500; Abcam, USA), followed by incubation with secondary biotinylated goat anti-rabbit/mouse IgG antibody (1:1000; Abcam, USA) for 30 min at 37°C, then stained by 0.02% 3,3′-diaminobenzidine (DAB) for 5 min (Boster, China), finally counterstained with hematoxylin. Negative controls were set by replacing the primary antibody with phosphate-buffered saline. The digital images were randomly taken at ×200 under light microscope (Olympus BX50, Japan) from four sections of each animal and analyzed by Image pro plus 6.0 (Media Cybemetics Inc., Silver Spring, MA, USA; Zhu et al., 2016). The data were expressed as mean density of positive area.

For immunofluorescence staining, the segments were fixed and embedded in optimal cutting temperature compound (OCT, Boster, China). Ten micrometer longitudinal cryo-sections were cut with freezing microtome (LEICA CM-1900, Germany; *n* = 3 rats/group), then permeabilized with 0.4% Triton X-100, incubated overnight at 4°C with rabbit anti-IL-4 (1:200; Abcam, USA), rabbit anti-IL-6 (1:1000; Abcam, USA), mouse anti-IL-13 (1:100; Abcam, USA), rabbit anti-TNF-α (1:500; Abcam, USA), rabbit anti-iNOS (1:100; Abcam, USA), mouse anti-CD68 (1:50; Abcam, USA), rabbit anti-arginase^−1^ (1:100; Abcam, USA), mouse anti-CD206 (1:50; Abcam, USA), mouse anti-vimentin (1:100; Abcam, USA), and mouse anti-S-100 (1:200; Abcam, USA), followed by incubation with anti-rabbit TRITC-IgG antibody (1:200; Abcam, USA) and anti-mouse FITC-IgG antibody (1:200; Abcam, USA) for 2 h at 37°C. Nuclei were stained with Hoechst33342 (1:200; Sigma, USA). The images were randomly taken at ×200 from four sections of each animal and the average fluorescent intensity was measured by Image pro plus 6.0 (Li et al., 2013). The expression intensity of the IL-4, IL-6, IL-13 and TNF-α were expressed as mean density of positive area. The number of M1 macrophages (iNOS^+^/CD68^+^) and M2 macrophages (arginase^−1+^/CD206^+^) was counted and expressed as a permillage of the total cells. Likewise the number of fibroblasts and SCs identified by vimentin and S-100 was counted respectively and expressed as a permillage of the total cells.

As it is well known that inflammatory responses are mainly occurred in the injured tissues and organs, the normalization with the contralateral sciatic nerve was not included in this experimental design.

### Assessment of Donor Cells Survival and Histopathology

At 1, 3, 6 and 9 weeks after bridging, the segments of the nerve were fixed and embedded in optimal cutting temperature compound. Ten micrometer longitudinal sections were obtained (*n* = 3 rats/group) for observing the survival and distribution of the donor GFP-cells. At 9 weeks postoperatively, the segments were embedded in paraffin and cut in 5 μm longitudinal sections (*n* = 3 rats/group). After stained with Hematoxylin and Eosin (H.E), the sections were used to observe for histopathological changes under a light microscope.

### Assessment of Functional Recovery

#### Lateral Foot Retraction Time (LFRT)

At 9 weeks, the sensory function recovery of the injured sciatic nerve was evaluated by right LFRT (*n* = 5 rats/group; Young et al., 2001). Right lateral foot were immersed in 50°C hot water bath respectively, then right LFRT for avoiding thermal damage was recorded.

#### Sciatic Function Index (SFI)

At 9 weeks, the motor function recovery of the injured nerve was evaluated by SFI (*n* = 5 rats/group; De Medinaceli et al., 1982; Varejão et al., 2001). The hind feet dipping in carbon ink were placed in a paper-lined walkway (10 × 80 cm) that led into a darkened cage. The footprints were measured according to the distance between the first and fifth toe (TS), second and fourth toe (ITS), the third toe and heel (PL). The experimental side was expressed as ETS, EITS and EPL, while the normal side was expressed as NTS, NITS and NPL, respectively. The SFI was calculated as follows: SFI = −38.3 (EPL − NPL)/NPL + 109.5 (ETS − NTS)/NTS + 13.3 (EITS − NITS)/NITS − 8.8. SFI value around −100 indicates total dysfunction, around 0 indicates normal nerve function.

#### Recovery Rate of Gastrocnemius Wet Weight

At 9 weeks, the gastrocnemius of rats were dissected from both sides after finishing electrophysiological analysis and weighed immediately (*n* = 5 rats/group). The wet weight on experimental side was expressed as a percentage of the normal side.

#### Electrophysiological Analysis

At 9 weeks, electrophysiological tests were performed by using a Haishen NDI-200P1 electroneurogram device (Shanghai, China; *n* = 5 rats/group; Pan et al., 2006, 2007). Under anesthetization, the previous surgical sites were re-exposed. Bipolar electrode was placed on the sciatic nerve proximal to the grafts and the comparable site on the contralateral side. The action potentials were recorded in the gastrocnemius with 0.5 mm precision measured by a sliding caliper. To ensure a maximum waveform and prevent independent muscle contractions, the stimulating intensity was 10 mA, the duration was 0.25 ms and the frequency was 1 Hz. Latency and wave amplitude were measured and calculated.

### Statistical Analysis

All values were expressed as mean ± standard deviation. Statistical differences were determined by using one-way factorial analysis of variance (ANOVA). Two-sample *t*-test was used to determine the difference between the groups. SPSS (version 22.0) statistical software was used for data analysis. *P* values less than 0.05 was considered statistically significant.

## Results

### Characteristics of EPI-NCSCs *In Vitro* and *In Vivo*

**Figure 2:**
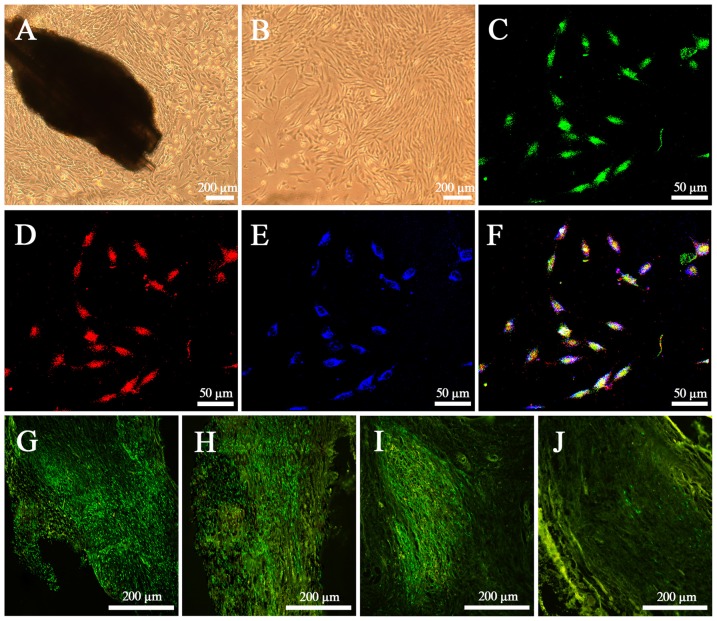
**Characteristics of epidermal neural crest stems cells (EPI-NCSCs) *in vitro* and *in vivo*. (A)** Morphology of EPI-NCSCs under inverted microscope on the 7th day. **(B)** Morphology of EPI-NCSCs under inverted microscope on the 11th day. **(C)** Expression of green fluorescence protein (GFP) (in green), **(D)** SOX10 (in red), **(E)** Nestin (in blue) and **(F)** merged on the 11th day. Distribution of green EPI-NCSCs at 1 week **(G)**, 3 weeks **(H)**, 6 weeks **(I)** and 9 weeks **(J)** after bridging respectively.

After culture for 72 h *in vitro*, EPI-NCSCs with round and spindle morphology started to emigrate onto the culture substratum from bulge explants, and continued to proliferate and began to become more elongated on 7th day (Figure 2A). After 4 days of rapid proliferation phase, the cells grew in clusters with the shape of a bipolar or a tripolar (Figure 2B). By identifying with the neural crest stem cell marker SOX10 (Figure 2D) and the progenitor cell marker Nestin (Figure 2E) on 11th day, GFP EPI-NCSCs (Figure 2C) were confirmed with purity up to 99% (Figure 2F). *In vivo*, EPI-NCSCs with green fluorescence were round and spindle morphology distributed along the nerve fibers in the nerve stumps, which could survive at least for 6 weeks (Figures 2G–I), but some green fluorescence cells could still be observed at 9 weeks after bridging (Figure 2J).

### EPI-NCSCs Changed the Expression Levels of Inflammatory Cytokines

**Figure 3:**
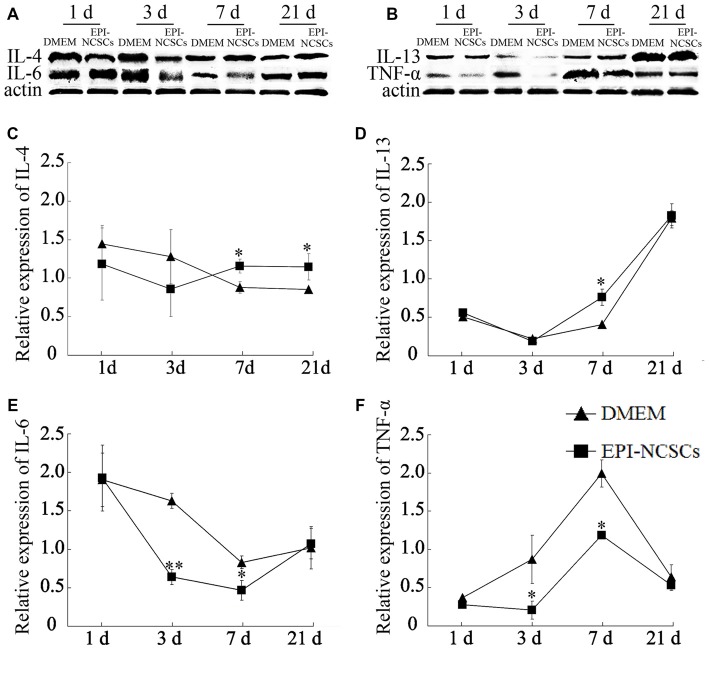
**Expression of inflammatory cytokines in the bridged sciatic nerve by western blot. (A)** Expression of IL-4 and IL-6. **(B)** Expression of IL-13 and TNF-α. **(C)** Relative expression of IL-4 by band intensity. **(D)** Relative expression of IL-13 by band intensity. **(E)** Relative expression of IL-6 by band intensity. **(F)** Relative expression of TNF-α by band intensity. *Values are significantly different at *P* < 0.05, compared with DMEM group; **Values are significantly different at* P* < 0.01, compared with DMEM group.

The cytokines expressions were determined at 1, 3, 7 and 21 days after bridging for observing the changes of local inflammation microenvironment. The expression levels of pro-inflammatory cytokines (IL-6 and TNF-α) and anti-inflammatory cytokines (IL-4 and IL-13) were determined by western blot (Figures 3A,B). The level of IL-4 in EPI-NCSCs group was significantly higher than the control group at 7 and 21 days (Figure 3C). The expression of IL-13 increased significantly at 7 days, but no difference between the groups at 21 days, as its expression was also increased in DMEM group (Figure 3D). IL-6 (Figure 3E) and TNF-α (Figure 3F) were expressed at significantly lower level in EPI-NCSCs group compared to DMEM group at 7 days, but no difference between the groups at 21 days.

**Figure 4:**
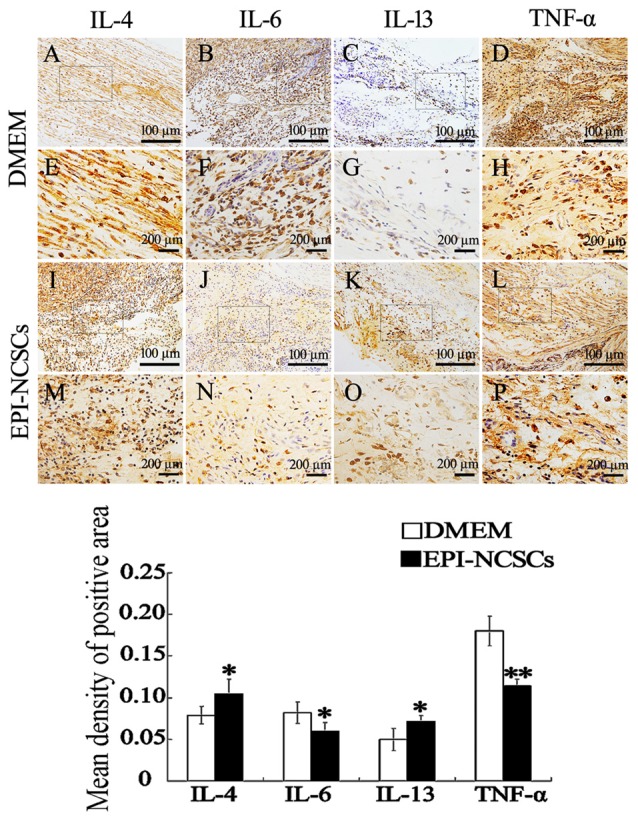
**Immunohistochemical staining of inflammatory cytokines in the nerve stump at 7 days after bridging. (E–H, M–P)** were enlarger of frames in **(A–D, I–L)**. *Values are significantly different at *P* < 0.05, compared with DMEM group; **Values are significantly different at* P* < 0.01, compared with DMEM group. **(A–D, I–L)**, scale bar = 100 μm. **(E–H, M–P)**, scale bar = 200 μm.

**Figure 5:**
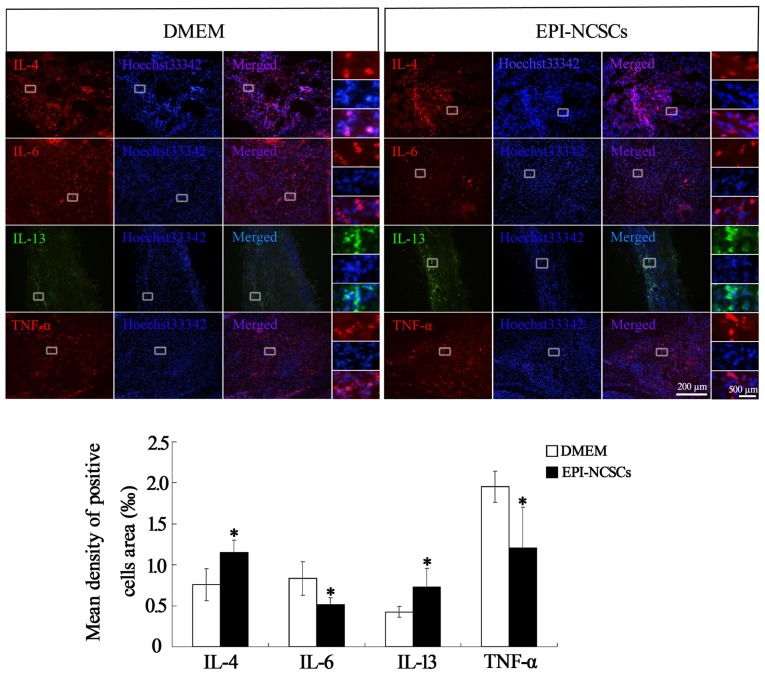
**Immunofluorescence staining of inflammatory cytokines in the nerve stump at 7 days after bridging**. *Values are significantly different at *P* < 0.05, compared with DMEM group. Scale bar = 200 μm. Enlarged inserts scale bars = 500 μm.

Pro-inflammatory cytokines and anti-inflammatory cytokines in the nerve stump were also observed by immunohistochemical (Figure 4) and immunofluorescence staining at 7 days after bridging (Figure 5), showing that the expression of IL-4 and IL-13 was remarkably enhanced. In contrast the expression of IL-6 and TNF-α was significantly weakened in EPI-NCSCs group compared with DMEM group.

### EPI-NCSCs Promoted the Transformation of Macrophages from Phenotype M1 to M2

**Figure 6:**
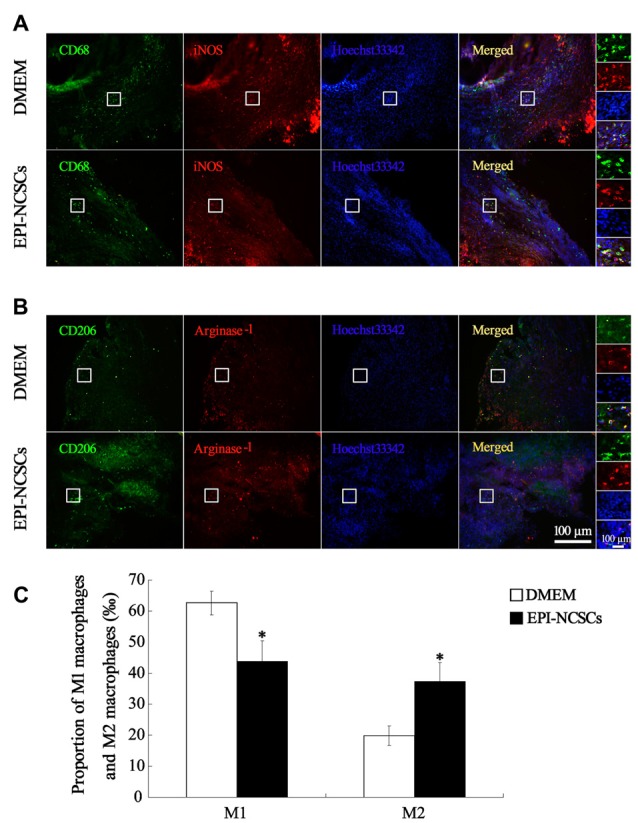
**Immunofluorescence staining showed M1 macrophages marked by CD68 and iNOS (A)** and M2 macrophages marked by CD206 and Arginase-1 **(B)** at 7 days after bridging **(C)** was the proportion of M1 macrophages and M2 macrophages. Scale bar = 100 μm.

In order to observe the polarization state of macrophages, the phenotypes of macrophages were identified by immunofluorescence staining of iNOS^+^/CD68^+^ for M1 macrophages and arginase^−1+^/CD206^+^ for M2 macrophages at 7 days after bridging. The total number of macrophages in EPI-NCSCs and DMEM group was 56.33 ± 5.78 and 51.42 ± 1.38, respectively, without statistical difference between two groups. However, the count of M1 macrophages in EPI-NCSCs group (28.33 ± 1.70) was significantly lower than that of DMEM group (44.08 ± 3.50, *P* < 0.01), but significantly higher for M2 macrophages in the former (23.08 ± 2.90) than in the latter (12.25 ± 2.63, *P* < 0.01). After the count of M1 and M2 macrophages was converted into permillage, M1 macrophages had a lower proportion in EPI-NCSCs group (43.84 ± 6.51‰) compared to DMEM group (62.62 ± 3.81‰, *P* < 0.05; Figures 6A,C). The proportion of M2 macrophages in EPI-NCSCs group (37.33 ± 6.08‰) was significant higher than that of DMEM group (19.87 ± 3.09‰, *P* < 0.05; Figures 6B,C). These indicated that EPI-NCSCs promoted the transformation of macrophages from Phenotype M1 to M2 at early phase.

### EPI-NCSCs Increased the Number of Myelin Forming SCs and Decreased the Number of Inflammatory Fibroblasts

**Figure 7:**
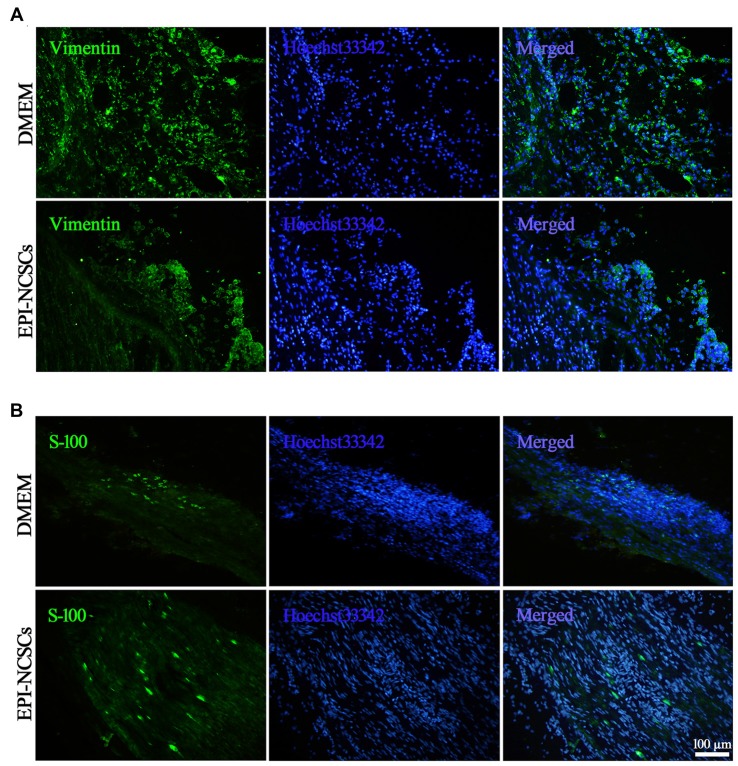
**Immunofluorescence staining showed fibroblasts (A)** and shwann cells (SCs) **(B)** marked by vimentin and S-100, respectively in the bridged sciatic nerve at 21 days. Scale bar = 100 μm.

For revealing the alterations of fibroblasts and myelin forming SCs following EPI-NCSCs transplantation as both of them playing different roles in the nerve repair, the former were identified by vimentin staining (Figure 7A), while the latter by S-100 staining (Figure 7B) at 21 days after bridging. Compared with DMEM group, the number of fibroblast was significantly decreased in the EPI-NCSCs group. As for SCs, their number was obviously increased in the distal stump of bridged site in EPI-NCSCs group compared to DMEM group (Table 1). The results showed that the number of fibroblasts was decreased, but increased for SCs number by EPI-NCSCs.

**Table 1:** **Number of fibroblasts and shwann cells (SCs) in the bridged sciatic nerve**.

	DMEM	EPI-NCSCs
Number of fibroblasts (‰)	69.40 ± 9.89	30.61 ± 4.88**
Number of SCs (‰)	26.71 ± 1.27	33.44 ± 2.94*

*Values are significantly different at P < 0.05; **Values are significantly different at P < 0.01.

### EPI-NCSCs Promoted the Recovery of Structure and Function of the Defected Sciatic Nerve

**Figure 8:**
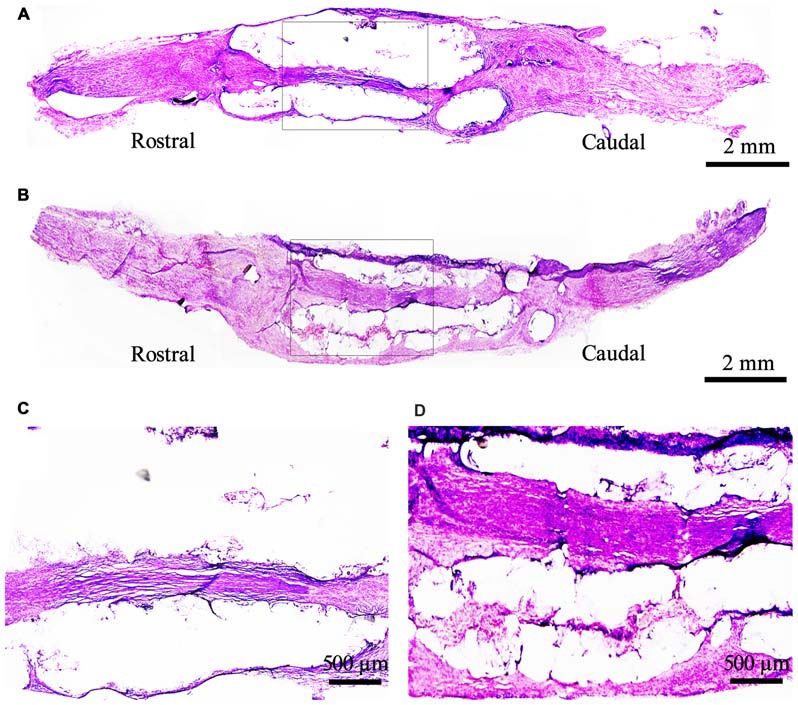
**Morphological structure of the bridged sciatic nerve by H.E staining under light microscope at 9 weeks. (A)** DMEM group; **(B)** EPI-NCSCs group; **(C, D)** Local magnification of **(A)** and **(B)** respectively. **(A,B)** scale bar = 2 mm. **(C,D)** scale bar = 500 μm.

**Figure 9:**
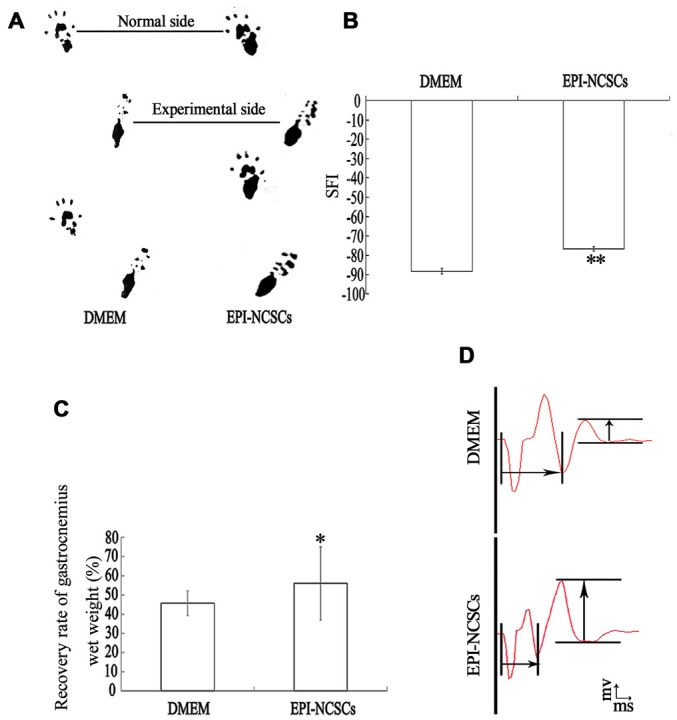
**Functional recovery of the bridged sciatic nerve at 9 weeks. (A)** Footprint; **(B)** sciatic function index; **(C)** recovery rate of gastrocnemius wet weight; **(D)** composite muscle action potential.

The structure and function of the defected nerve were assessed by morphological observation and different measuring methods at 9 weeks to determine whether EPI-NCSCs were effective to improve both of them. Under light microscope, the tissue organization of the injured nerve was distinctive between EPI-NCSCs (Figures 8B,D) and DMEM group (Figures 8A,C). In the former, the tissue structure was more compact and the interstitial space was smaller, while more loose structure and larger spaces could be observed in the later. LFRT was significantly shortened (2.45 ± 0.47 s) in EPI-NCSCs group compared to DMEM group (3.19 ± 0.18 s, *P* < 0.05). Compared with the DMEM group (−88.31 ± 1.39), the TS and ITS became longer, and the PL was more shorter in the EPI-NCSCs group (−77.81 ± 1.06, *P* < 0.01, Figures 9A,B). Although gastrocnemius suffered in both groups, recovery rate of gastrocnemius wet weight with EPI-NCSCs (56.02 ± 4.27%) was higher than that with DMEM (45.71 ± 6.40%, *P* < 0.05, Figure 9C). Electrophysiological examination indicated that the latency was 2.80 ± 0.33 ms and the amplitude was 2.04 ± 0.80 mV in EPI-NCSCs group, which was much better than 5.20 ± 1.20 ms and 0.50 ± 0.37 mV in DMEM group (*P* < 0.01, Figure 9D). All of these indexes indicated that EPI-NCSCs could improve the structure and function of defected nerve.

## Discussion

In the present study, the defected sciatic nerve of rats was used to investigate the effects of EPI-NCSCs on local inflammation microenvironment expressed by pro- and anti-inflammatory cytokines, as well as activation of inflammatory cells. At the same time, the changes of sciatic nerve structures and function were determined. The results showed that EPI-NCSCs could regulate the dynamic balance of pro- (IL-6 and TNF-α) and anti-inflammatory (IL-4 and IL-13) cytokines after bridging, promote the transformation of macrophages from M1 to M2, and increase myelin-forming SCs, but decrease scar-forming fibroblasts. These results could be related to improvement of structures and function of the defect nerve.

It has been reported previously that EPI-NCSCs can directly or indirectly influence expression and secretion of numerous neurotrophic factors such as nerve growth factor (NGF), brain-derived neurotrophic factor (BDNF), fibroblast growth factors (FGF), insulin-like growth factor-1 (IGF-1; Hu et al., 2010), transforming growth factor-β (TGF-β) and their cognate receptors (Ren et al., 2001; Hu et al., 2006). Recently, studies have focused on the potential of EPI-NCSCs to provide neuroprotection for damaged neurons, stimulate local axonal sprouting, and induce or facilitate neovascularization (Kocsis, 2009). Therefore the transplanted EPI-NCSCs can promote the structure and function to recover after central (Amoh et al., 2008; Sieber-Blum, 2010; Liu et al., 2011) and peripheral nerve injury (Amoh et al., 2011). Here, it was further demonstrated that whether these recoveries were also related to the regulatory effects of EPI-NCSCs on local inflammation microenvironment besides their biological effects mentioned above.

As a part of the initial response to the sciatic nerve injury (Chen et al., 2011), pro-inflammatory cytokines were up-regulated during the acute phase typically lasting for a few hours, which can augmentate tissue damage, neuronal and axonal destruction and demyelination close to the injury site (Pourgonabadi et al., 2016). Between 2 and 7 days after injury, anti-inflammatory cytokines of the sub-acute phase start to increase, whereas pro-inflammatory cytokines decrease with the activation of inflammatory cells (Beck et al., 2010). In the chronic phase, lasting for weeks or even months, the injury sites are cleaned by the development of specific humoral and cellular immune responses (Donnelly and Popovich, 2008), which finally support sciatic nerve regeneration. The present results showed that after EPI-NCSCs transplantation, pro-inflammatory cytokines, IL-6 and TNF-α, were increased at the first stage, then decreased gradually with the time prolonging. In contrast, anti-inflammatory cytokines, IL-4 and IL-13, were initially lower, but significantly higher at later stages. This increase may be related to the polarization of M2 macrophages and down-regulation of IL-6 and TNF-α (Takami et al., 2002; Moghaddam et al., 2015). The pro-inflammatory cytokines increased in the acute phase can induce the expression of chemokines and gather inflammatory cells to cause the neuronal damage (Vidal et al., 2013). Their decreased expression at later stages may involve in the removal of necrotic tissue and promote reconstruction of the nerve (Joo et al., 2012). The enhanced expression of anti-inflammatory cytokines during sub-acute phase is important to elucidate the polarization and function of macrophages (Martinez et al., 2008).

Macrophages are highly differentiated and mature mononuclear phagocytic cell and exert different functions depending on the microenvironment (Nadeau et al., 2011). During PNI and consequent inflammation, macrophages are supposed to antagonize the inflammation (Pasterkamp et al., 1999; Miller et al., 2004; Ji et al., 2009). Among macrophages, phenotype M1 induced by pro-inflammatory cytokines such as IL-6 and TNF-α can promote oxidative metabolism and damage neurons. M2 macrophages induced by anti-inflammatory cytokines such as IL-4 and IL-13 can inhibit pro-inflammatory responses, improve angiogenesis, and promote the regeneration of mature sensory axons (Higuchi et al., 2016; Zhang et al., 2017). Likewise, this study demonstrated that EPI-NCSCs could increase the number of M2 macrophages and decrease the number of M1 macrophages at 7 days after transplantation, which was consistent with the change of pro- and anti-inflammatory cytokines. These results suggested that EPI-NCSCs could promote towards polarization of macrophages anti-inflammatory M2 phenotypes to provide a neuroprotective microenvironment.

Activated fibroblasts in the injured site can regulate inflammation during the repair of tissue damage by generating pro-inflammatory cytokines transiently (Ara et al., 2009; Konermann et al., 2012). Within the chronic inflammation phase, the fibroblasts remain active and aggravate the inflammation. The inflammatory signal can be further amplified and spur pro-inflammatory activation with the secretion of CCL_3_ by fibroblasts (Burdick et al., 1993). The present results showed that EPI-NCSCs could decrease the number of fibroblasts at 21 days after bridging. These may contribute in reducing the excessive activation of fibroblasts and regulate the inflammation microenvironment, which are beneficial to the repair of defected sciatic nerve (Ibba-Manneschi et al., 2016; Kunanusornchai et al., 2016; Morin and Grenier, 2017).

SCs are neural crest-derived glial cells that support axons of the peripheral nerves (Zhan et al., 2015). Following PNI, are contributed to the removal of myelin debris, undergo dedifferentiation, proliferation and migration to form Büngner band, thus guide the growth of regenerating axons toward the denervated targets (Namgung, 2014). Inducing SCs differentiated to myelin-formed SCs and promoting their migration in the injury region are the key point for the repair of PNI (Stoll et al., 1989). It was showed in the present results that the number of myelin-formed SCs was increased by EPI-NCSCs. Although nerve tracing was not performed in this study as it has been confirmed previously by Li B. C. et al. (2010) and You et al. (2011) and others (Wang and Zhang, 2014; Nadal-Nicolás et al., 2015; Agbaje et al., 2016; Guimarães et al., 2016), increased SCs are certainly beneficial to the regeneration and the growth of injured axons.

In the present study, recovery of the structure and the function of the bridged nerve with EPI-NCSCs was significantly superior to the DMEM as confirmed by the histopathological observation and by the comparison of LFRT, SFI, gastrocnemius wet weight and electrophysiology. Importantly, EPI-NCSCs could at least survive in the bridged nerve for 9 weeks. Taking these with the changing features of cytokines, macrophages, fibroblasts and SCs together, it was suggested that EPI-NCSCs could be able to promote the repair of structure and function of defected sciatic nerve by improving local inflammation and regeneration microenvironment, but it requires further proof. Besides, the relationship between inflammation microenvironment and nerve fiber regeneration should also need to be studied in the future.

## Author Contributions

YL and DY: surgical procedures, tissue collection, behavioral measurements, staining, western blots, data analysis, figure preparation, data discussion, and wrote the manuscript. JZ: contributed in data discussion and in writing the manuscript. BL: contributed in experimental design, data discussion. LZ: contributed in surgical procedures. HF and BCL: directed the project, experimental design, discussion, responsible for revising the manuscript.

## Conflict of Interest Statement

The authors declare that the research was conducted in the absence of any commercial or financial relationships that could be construed as a potential conflict of interest.
